# Genome-wide detection of CNV regions and their potential association with growth and fatness traits in Duroc pigs

**DOI:** 10.1186/s12864-021-07654-7

**Published:** 2021-05-08

**Authors:** Yibin Qiu, Rongrong Ding, Zhanwei Zhuang, Jie Wu, Ming Yang, Shenping Zhou, Yong Ye, Qian Geng, Zheng Xu, Sixiu Huang, Gengyuan Cai, Zhenfang Wu, Jie Yang

**Affiliations:** 1 College of Animal Science and National Engineering Research Center for Breeding Swine Industry, South China Agricultural University, Guangzhou, Guangdong 510642 People’s Republic of China; 2 Guangdong Wens Breeding Swine Technology Co., Ltd., Yunfu, Guangdong 527400 People’s Republic of China; 3 Lingnan Guangdong Laboratory of Modern Agriculture, Guangzhou, 510642 People’s Republic of China

**Keywords:** Copy number variation, CNVR-based GWAS, Growth, Fatness, Duroc pigs

## Abstract

**Background:**

In the process of pig breeding, the average daily gain (ADG), days to 100 kg (AGE), and backfat thickness (BFT) are directly related to growth rate and fatness. However, the genetic mechanisms involved are not well understood. Copy number variation (CNV), an important source of genetic diversity, can affect a variety of complex traits and diseases and has gradually been thrust into the limelight. In this study, we reported the genome-wide CNVs of Duroc pigs using SNP genotyping data from 6627 animals. We also performed a copy number variation region (CNVR)-based genome-wide association studies (GWAS) for growth and fatness traits in two Duroc populations.

**Results:**

Our study identified 953 nonredundant CNVRs in U.S. and Canadian Duroc pigs, covering 246.89 Mb (~ 10.90%) of the pig autosomal genome. Of these, 802 CNVRs were in U.S. Duroc pigs with 499 CNVRs were in Canadian Duroc pigs, indicating 348 CNVRs were shared by the two populations. Experimentally, 77.8% of nine randomly selected CNVRs were validated through quantitative PCR (qPCR). We also identified 35 CNVRs with significant association with growth and fatness traits using CNVR-based GWAS. Ten of these CNVRs were associated with both ADG and AGE traits in U.S. Duroc pigs. Notably, four CNVRs showed significant associations with ADG, AGE, and BFT, indicating that these CNVRs may play a pleiotropic role in regulating pig growth and fat deposition. In Canadian Duroc pigs, nine CNVRs were significantly associated with both ADG and AGE traits. Further bioinformatic analysis identified a subset of potential candidate genes, including *PDGFA*, *GPER1*, *PNPLA2* and *BSCL2*.

**Conclusions:**

The present study provides a necessary supplement to the CNV map of the Duroc genome through large-scale population genotyping. In addition, the CNVR-based GWAS results provide a meaningful way to elucidate the genetic mechanisms underlying complex traits. The identified CNVRs can be used as molecular markers for genetic improvement in the molecular-guided breeding of modern commercial pigs.

**Supplementary Information:**

The online version contains supplementary material available at 10.1186/s12864-021-07654-7.

## Background

Genetic variation occurs in many forms, including single nucleotide polymorphisms (SNPs), insertions/deletions (INDELs) of small genetic fragments, and copy number variations (CNVs), in human and animal genomes. CNVs are a particular subtype of genomic structural variation that range from approximately 50 bp to several Mb and are mainly represented by deletions and duplications [1–4]. Adjacent copy number variation areas with overlapping regions can be combined into a large genome segment, known as the copy number variation region (CNVR) [5]. In terms of the total bases involved, CNVs encompass more nucleotide sequences and arise more frequently than SNPs [6]. Therefore, they have higher mutation probability and more significant potential impacts [7], such as changing gene structure and altering gene dosage and thus dramatically affect gene expression and adaptive phenotypes [8]. Additionally, some CNVs are associated with several complex diseases [9–11]. These observations led us to predict that CNVs are a primary contributor to phenotypic variation and disease susceptibility.

Indeed, multiple studies have suggested that CNVs play an essential role in affecting some complex traits and causing disease. In humans, Aitman et al. [12] demonstrated that copy number polymorphism in the *Fcgr3* gene is a determinant of susceptibility to immunologically mediated renal disease; additionally, a recent study identified that copy number variation in *NPY4R* might be related to the pathogenesis of obesity [13]. Similarly, phenotypic variations and diseases caused by CNVs are also widespread in domesticated animals. For example, in pigs, the focus of this study, an increase in copy number (CN) of the *KIT* gene is associated the dominant white phenotype [14, 15]. With regard to reproductive performance, CNV in the *MTHFSD* gene was reportedly correlated with litter size in Xiang pigs [16]. Zheng et al. [17] also showed that a higher CN of the *AHR* gene had a positive effect on litter size. With regard to productive performance, Revilla et al. [18] discovered a CNVR containing the *GPAT2* gene, which might be associated with several growth-related traits. Thus, analyzing CNVs and identifying their potential association with complex traits has gradually become an essential part of genetic studies.

Growth rate and fatness are vital objectives in the process of pig breeding, and are directly associated with economic advantages. The growth rate measured at different stages mainly include average daily gain (ADG) during the test period as well as with age (AGE), which was defined as estimated age at a certain weight [19]. Fat deposition is also a critical biological process that is generally measured as the backfat thickness (BFT). Until now, considerable association analysis has focused on identifying single-site variants, quantitative trait loci (QTLs), and related candidate functional genes that might influence growth and fatness traits [20–22]. However, systematic association studies of complex quantitative traits based on CNVs have rarely been conducted [18, 23], and the full relevance of CNVs to the genetic basis of these traits is yet to be clarified. In addition, the genetic architecture of these traits is complex and usually controlled by multiple genes [19]. The majority of association studies for growth and fatness traits in pigs have used only a small number of genotyped animals, which has limited the statistical power of the association analysis [24]. It is therefore necessary to conduct CNV association analysis in a population with a sufficiently large sample size.

In this study, we performed genome-wide CNV detection in a large population of Duroc pigs of U.S. and Canadian origin. Moreover, CNVR-based genome-wide association studies (GWAS) of growth and fatness traits were applied to the two experimental populations. We identified CNVR and candidate genes that can provide additional information on the molecular mechanisms underlying important economic traits and promote the rapid development of molecular breeding approaches in pigs.

## Results

### Detection of genome-wide CNVs in two pig populations

**Fig. 1:**
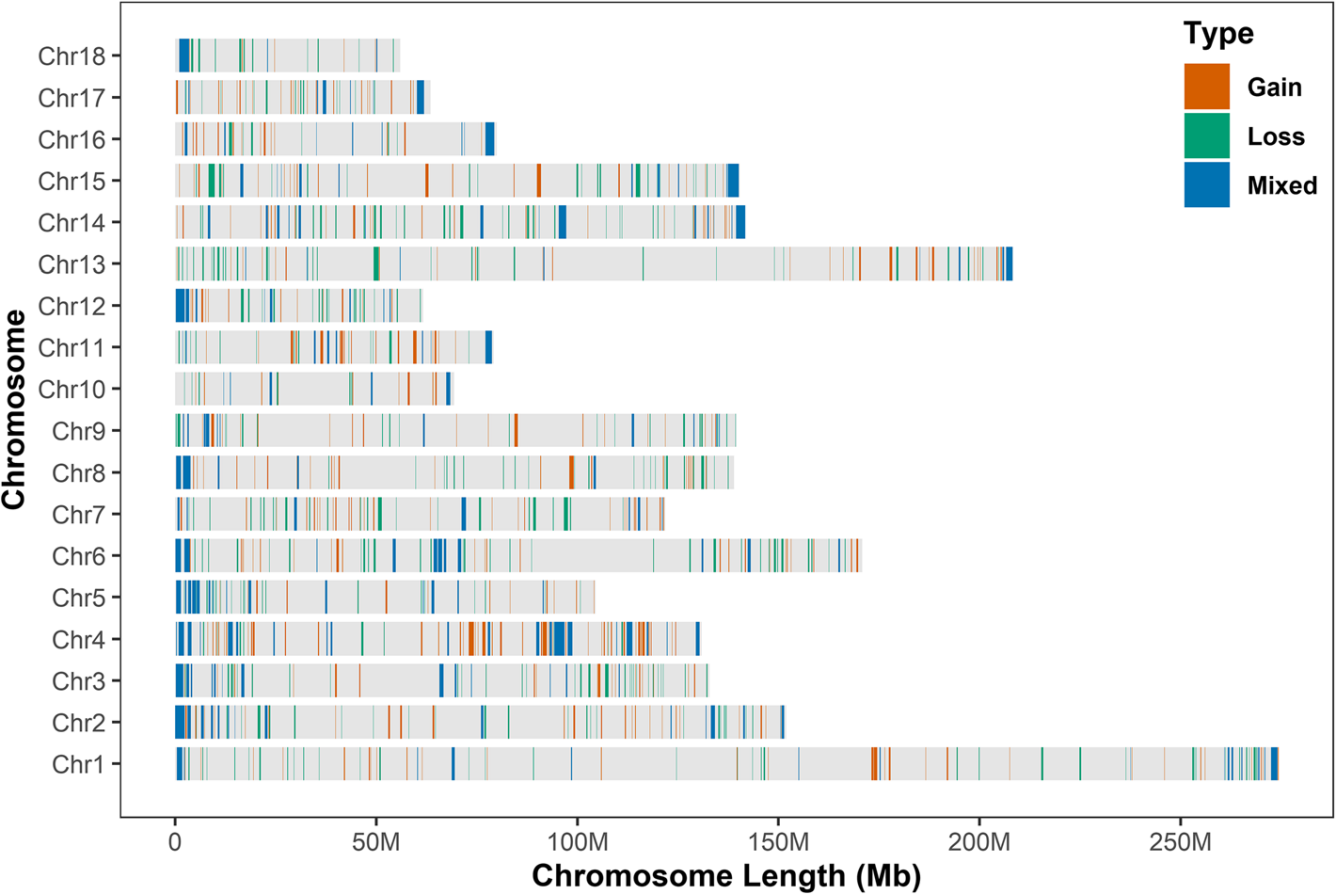
The overall CNVR maps for U.S. and Canadian Duroc pigs in the 18 autosomes. Three types of CNVR are identified, including gain (red), Loss (green), and Mixed (blue). Y-axis values are autosomes, and X-axis values are chromosome position in Mb

We detected CNVs in 18 autosomes in Duroc pigs of Canadian and U.S. origin using PennCNV software v1.0.5 [25]. A total of 33,347 CNVs (5403 losses and 27,944 gains) were identified in 5928 pigs. Among these, 19,987 CNVs were from 3271 Duroc pigs of U.S. origin, and 13,360 CNVs were from 2657 Duroc pigs of Canadian origin. These CNVs were merged to identify CNVRs (see Additional file 1: Table S1). A total of 953 CNVRs were identified in the two populations with 388 gains, 376 losses, and 189 mixed variations (gains and losses occurring in the same region). Table 1 and the CNVR map (Fig. 1) summarize the distribution of total CNVRs on different autosomes. CNVRs in chromosome 4 (SSC4) had the highest coverage (20.64%) while those in SSC1 had the lowest (6.43%). The number of CNVRs varied from 20 (SSC18) to 82 (SSC1), and the total size of CNVRs detected in this study was 246.89 Mb, accounting for ~ 10.90% of the pig autosomal genome.

**Table 1:** Chromosome distribution of all 953 CNVRs in the pig autosomes

Chr	Chr length (kb)	CNVR counts	Length of CNVR (kb)	Coverage (%)	Max size (kb)	Average size (kb)	Min size (kb)
1	274,330.53	82	17,626.89	6.43	1592.58	130.23	11.81
2	151,935.99	66	18,291.80	12.04	2380.53	170.95	22.54
3	132,848.91	62	15,208.53	11.45	1909.62	165.13	22.47
4	130,910.91	75	27,024.95	20.64	2599.17	202.77	16.65
5	104,526.01	47	11,743.52	11.24	1237.96	162.43	32.04
6	170,843.59	68	19,996.87	11.70	1410.90	178.36	24.07
7	121,844.10	59	14,085.72	11.56	1037.19	166.25	18.22
8	138,966.24	53	12,195.85	8.78	1917.30	129.15	31.23
9	139,512.08	50	11,559.95	8.29	900.56	164.24	26.12
10	69,359.45	22	5184.21	7.47	1036.82	159.50	10.40
11	79,169.98	37	10,437.79	13.18	1635.59	168.97	40.53
12	61,602.75	40	10,988.12	17.84	2225.46	185.49	21.53
13	208,334.59	71	15,199.02	7.30	1662.75	139.99	24.59
14	141,755.45	75	18,455.58	13.02	2234.96	145.26	23.66
15	140,412.72	55	16,181.67	11.52	2721.56	147.32	24.13
16	79,944.28	31	8505.51	10.64	2187.75	174.40	29.21
17	63,494.08	40	8183.03	12.89	1822.61	124.68	48.59
18	55,982.97	20	6020.53	10.75	2495.98	168.20	29.92

**Fig. 2:**
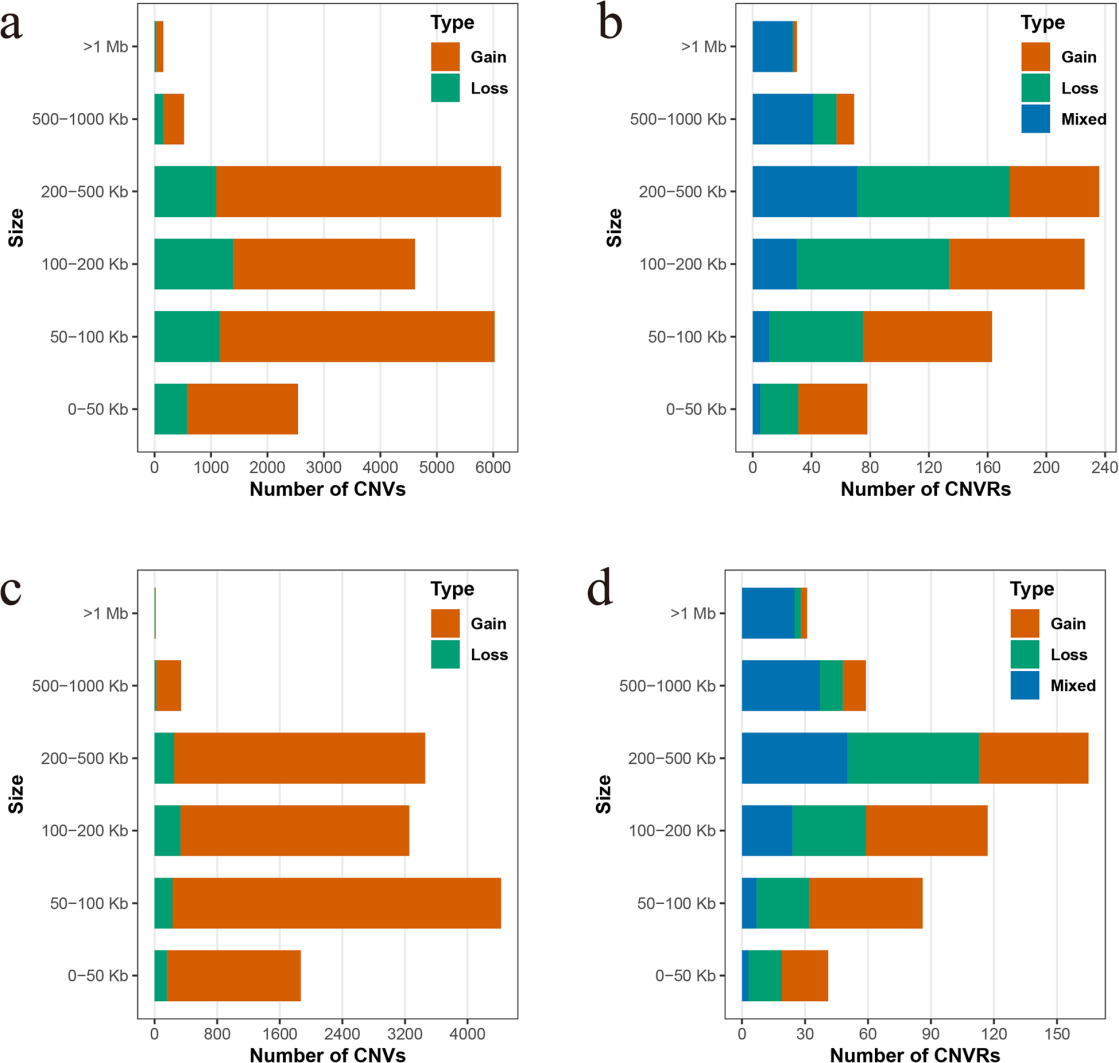
CNV and CNVR distribution of U.S. and Canadian Duroc pigs according to the size interval. The plots of (**a**) and (**b**) show the CNV and CNVR distribution in U.S. Duroc pigs, respectively. The plots of (**c**) and (**d**) show the CNV and CNVR distribution in Canadian Duroc pigs, respectively

By matching the CNVs in each population to the corresponding CNVRs, we identified 802 CNVRs in the U.S. Duroc pigs, 499 CNVRs in the Canadian Duroc pigs, with 348 CNVRs that were shared by both populations (see Additional file 2: Table S2). CNVs in U.S. Duroc pigs ranged in size from 10.4 kb to 2.6 Mb, averaging 183.6 kb (Fig. 2a), while CNVR size ranged from 10.4 kb to 2.7 Mb (Fig. 2b). In Canadian Duroc pigs, CNV size ranged from 10.4 kb to 2.1 Mb, with an average of 165.2 kb (Fig. 2c), while CNVR size ranged from 10.4 kb to 2.7 Mb (Fig. 2d). In summary, most CNVs and CNVRs in both populations were 50–500 kb in size, with the CNVRs covering ~ 9.56 and 7.44% of the porcine genome (*Sus scrofa* 11.1) in U.S. and Canadian Duroc pigs, respectively. Notably, CNV duplications were more likely to occur in both populations. In addition, we found that among the top 20 largest CNVRs, 19 were mixed types. More intriguingly, 15 of them (75%) were resided in telomeric regions (Fig. 1), indicating that CNVs occur more frequently towards telomeres, which are hot spots for the recombination and duplication of large fragments [26].

### Comparison of CNVRs detected in previous swine studies

We compared the CNVRs identified in this study with those in nine previous swine studies based on Scrofa11.1 assembly (see Additional file 3: Table S3). For CNVRs based on the early porcine assembly 10.2, we converted the data to Scrofa11.1 assembly using the UCSC LiftOver tool (http://genome.ucsc.edu/cgi-bin/hgLiftOver). The results show varying levels of overlapping CNVRs in the studies (Table 2), due to differences in breed, platform, algorithm, and CNV definition, which significantly impact the results [33]. We used a much looser definition of overlap, where two CNVRs were considered to overlap as long as they shared at least one nucleotide base [34].

**Table 2:** Comparison of CNVRs identified in this study with other studies (based on the Sscrofa 11.1 genome assembly)

Study	Platform	Software	Breeds (Number^**1**^)	Samples	Number of CNVRs^**2**^ (original CNVRs^**3**^)	Number of overlapped CNVRs in this study
Chen et al. [27]	Porcine SNP60	PennCNV	Duroc, Rongchang, etc. (18)	1693	243 (565)	69
Wang et al. [28]	Porcine SNP60	PennCNV	Duroc, Laiwu, etc. (10)	302	146 (348)	37
Wiedmann et al. [29]	Porcine SNP60	PennCNV	α Mixed Breed Swine (1)	1802	185 (502)	37
Wang et al. [30]	1 M aCGH	Agilent Genomic Workbench	Duroc, Yorkshire, etc. (9)	12	436 (758)	44
Xie et al. [31]	Porcine SNP60	PennCNV	Xiang, Kele (2)	120	75 (172)	15
Stafuzza et al. [32]	Porcine SNP80	PennCNV	Duroc (1)	3520	136 (425)	81
Wang et al. [33]	Porcine SNP80	PennCNV	Large White (1)	857	175 (312)	97
Keel et al. [34]	Next-generation sequencing	CNVnator & LUMPY	Duroc, Landrace, etc. (3)	240	3538	338
Zheng et al. [17]	Next-generation sequencing	CNVnator & CNVcaller	Duroc (1)	29	6700	1030

All CNVRs identified in other studies were converted to Sscrofa 11.1 genome assembly using the liftOver tool. ^1^Pig breeds used for comparison; ^2^Successfully converted CNVRs; ^3^Original number of CNVRs

The most considerable overlap in CNVRs identified between this study and previous studies was observed with results obtained from next-generation sequencing platforms (see Additional file 3: Table S3). The percentages of overlapped CNVRs were 21.72 and 21.82%, respectively [17, 34].

### Validation of identified CNVRs using qPCR

**Fig. 3:**
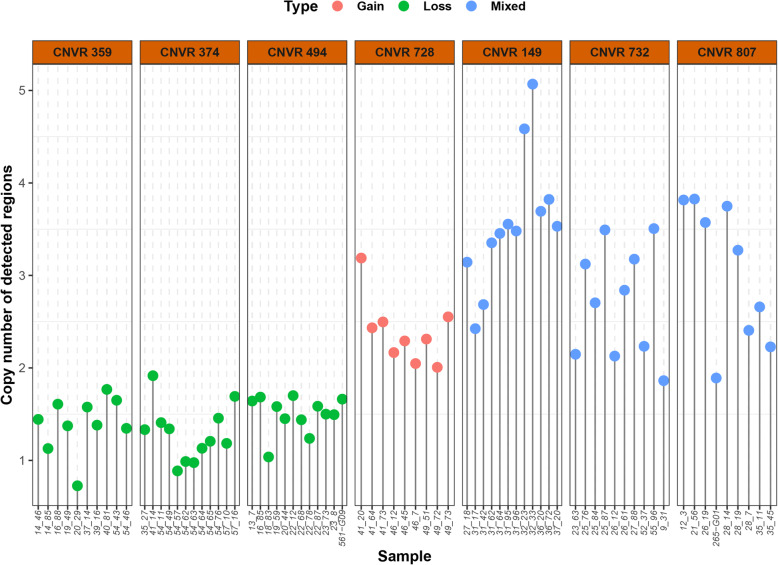
The results of qPCR validation in selected CNVRs. The x-axis represents the tested sample ID. The y-axis represents different copy number. Values of approximately 2 were considered normal. A value of 3 or more and a value of 1 or less represented gain and loss statuses, respectively

To confirm the reliability of the identified CNVRs, we randomly selected nine CNVRs (CNVR 149, 359, 374, 494, 621, 728, 732, 807, and 878) that co-localized with the *ELFN1*, *PUSL1*, *MAPRE2*, *SGMS2*, *PCID2*, *DSCAM*, *GATD3A ADGRA1*, and *LIFR* genes, respectively. Seven of these CNVRs (CNVR 149, 359, 374, 494, 728, 732, and 807) were successfully validated (Fig. 3). Details of the primers used are listed in Additional file 4: Table S4.

### CNVR frequency in two Duroc pig populations

**Fig. 4:**
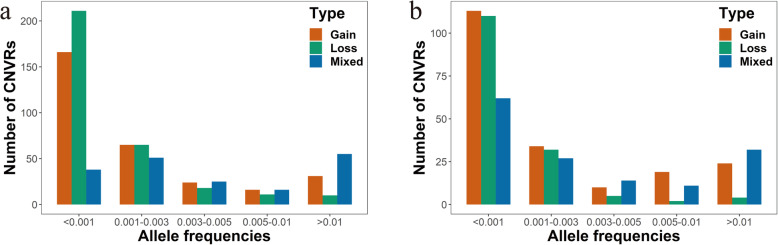
The allele frequencies of CNVRs in the U.S. (**a**) and Canadian Duroc (**b**) pigs

We also calculated the frequencies of the CNVRs in the U.S. (Fig. 4a) and Canadian (Fig. 4b) Duroc pig populations. The frequency of CNVR in U.S. Duroc pigs varied from 0.030% (detected in one pig) to 40.6% (1327 of 3271 pigs). In the Canadian Duroc pigs, CNVR frequencies ranged from 0.038% (detected in one pig) to 52.2% (1386 of 2657 pigs). Moreover, the frequency of CNVRs was concentrated at 0.03–0.3%, indicating most CNVRs are rare, only exist in a few animals and are challenging to measure reliably [35]. For this reason, CNVR-based GWAS were performed using CNVRs with frequencies exceeding 0.5% [32].

### Phenotypic and CNVR-based GWAS statistics

To further characterize the functions of CNVRs in pigs, GWAS were performed for three quantitative traits. The statistical summaries of ADG, AGE, and BFT in the two populations are listed in Table 3. All phenotypic data approximately followed a normal distribution.

**Table 3:** The statistics for the phenotypes of growth traits and fatness in two pig populations

Population	Trait^**1**^	Unit	N^**2**^	Mean(±SD)^**3**^	Min^**4**^	Max^**5**^	C.V.(%)^**6**^
U.S. Duroc	ADG	g/day	3292	619.36 ± 31.76	525.61	716.58	5.13
	AGE	day	3292	158.99 ± 8.21	134.42	182.70	5.16
	BFT	mm	3276	8.9 ± 0.95	6.09	12.27	10.67
Canadian Duroc	ADG	g/day	2595	611.92 ± 42.16	483.55	738.4	6.89
	AGE	day	2592	161.13 ± 11.15	127.82	195.29	6.92
	BFT	mm	2574	9.55 ± 1.77	5.1	15.06	18.53

^1^*ADG* Average daily gain at 100 kg; *AGE* Days to 100 kg; *BFT* Backfat thickness at 100 kg; ^2^Number of animals (N); ^3^Mean (standard deviation); ^4^Minimum (min); ^5^Maximum (max); ^6^Coefficient of variation (C.V.)

**Fig. 5:**
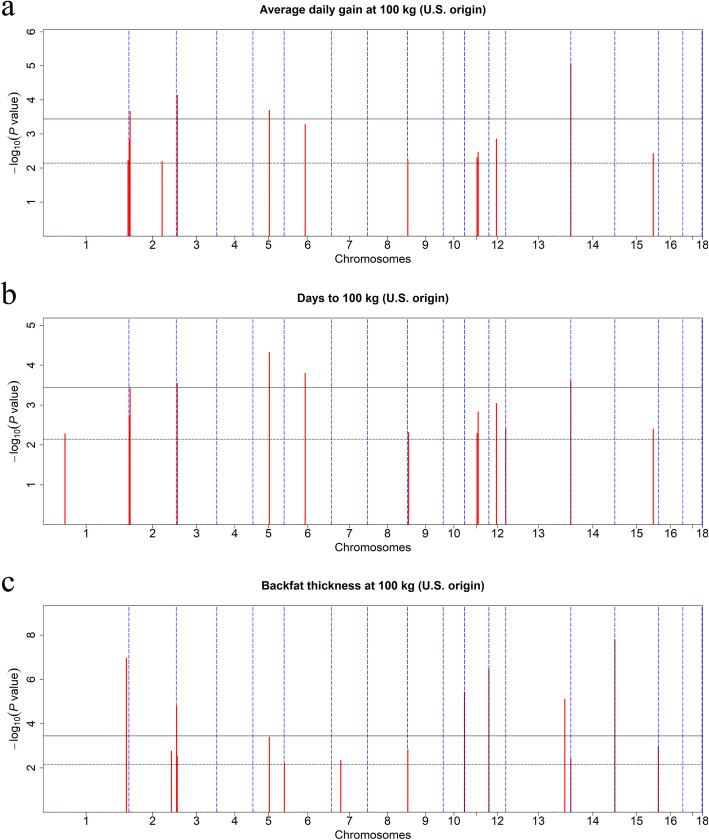
Manhattan plots of CNVR-based GWAS in the U.S. Duroc pig population. Manhattan plots consisted of average daily gain at 100 kg (**a**), days to 100 kg (**b**), and backfat thickness at 100 kg (**c**). The x-axis represents the chromosomes, and the y-axis represents the -log10(*P*-value). The solid and dashed lines indicate the 5% genome-wide (3.60E-04) and suggestive (7.19E-03) Bonferroni-corrected thresholds, respectively

**Fig. 6:**
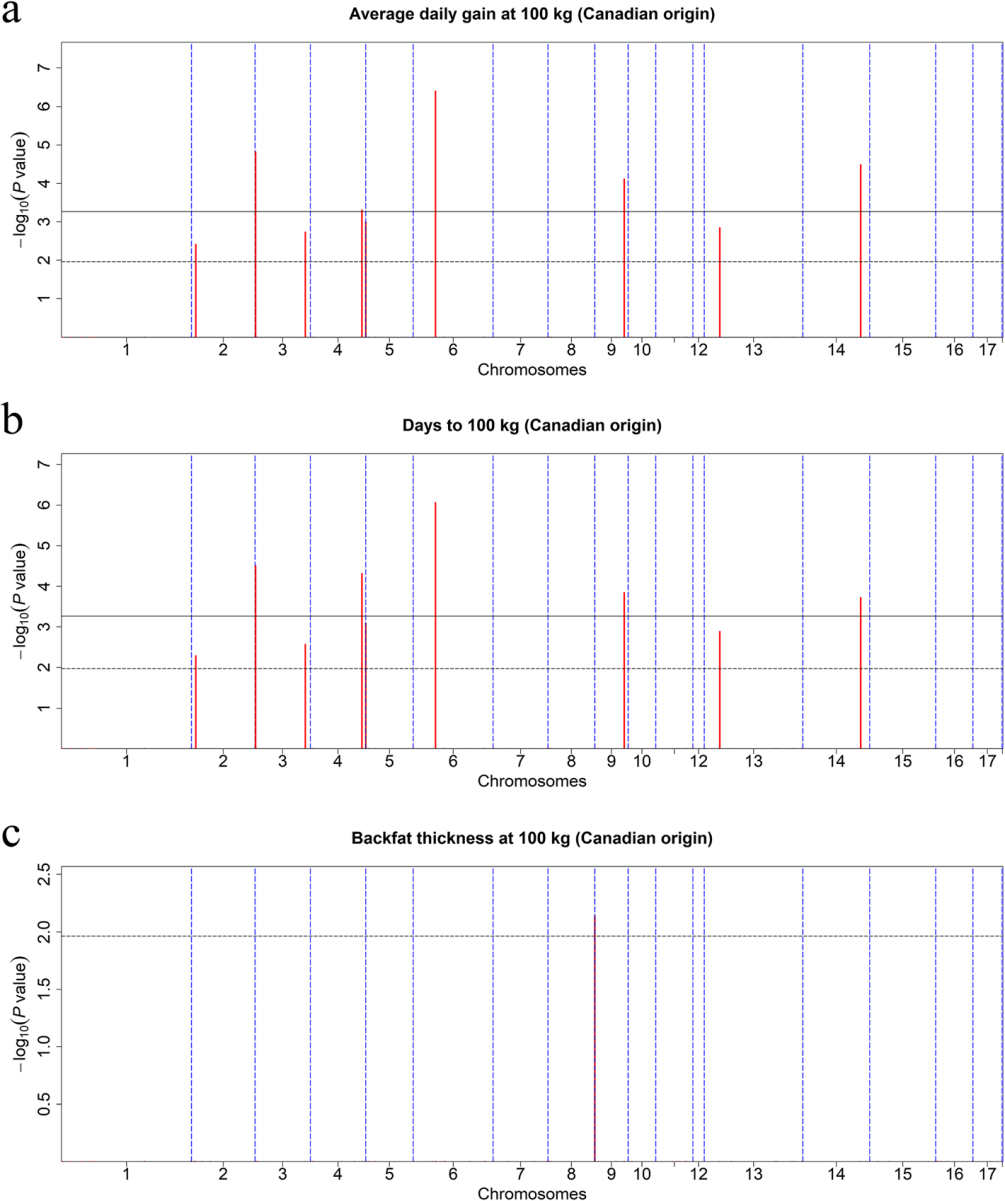
Manhattan plots of CNVR-based GWAS in the Canadian Duroc pig population. Manhattan plots consisted of average daily gain at 100 kg (**a**) and days to 100 kg (**b**), and backfat thickness at 100 kg (**c**). The x-axis represents the chromosomes, and the y-axis represents the -log10(*P*-value). The solid and dashed lines indicate the 5% genome-wide (5.43E-04) and suggestive (1.09E-02) Bonferroni-corrected thresholds, respectively

Since most CNVRs have a low frequency that is challenging to measure reliably, we used CNVRs with frequencies higher than 0.5% in each population for further analysis, to improve the reliability of the GWAS results [32]. A total of 139 CNVRs from 3303 U.S. Duroc pigs and 92 CNVRs from 2677 Canadian Duroc pigs were selected for association analysis. The Manhattan plots and significant CNVRs obtained from separate association analyses in these two populations are shown in Figs. 5 and 6, Tables 4 and 5.

**Table 4:** Significant CNVRs associated with growth traits in U.S. and Canadian Duroc pigs

Population	Traits^**1**^	CNVR ID^**2**^	Type^**3**^	Chromosome	Start (bp)	End (bp)	***P***-value^**4**^	Candidate genes
U.S. Duroc	ADG	CNVR 79	Gain	1	270,413,118	270,492,246	6.07E-03	
	ADG	CNVR 122	Gain	2	105,908,671	106,135,493	6.39E-03	
	ADG	CNVR 514	Loss	9	619,847	1,268,010	5.71E-03	
	ADG; AGE	**CNVR 83**	Mixed	2	27,459	2,407,984	1.54E-03; 1.78E-03	*PNPLA2*
	ADG; AGE	**CNVR 85**	Mixed	2	3,094,575	3,946,741	**2.16E-04**; 3.67E-04	
	ADG; AGE	**CNVR 152**	Mixed	3	2,789,839	3,480,462	**7.38E-05**; **2.85E-04**	*SDK1*
	ADG; AGE	**CNVR 315**	Gain	5	52,276,104	52,737,350	**2.01E-04**; **4.78E-05**	
	ADG; AGE	**CNVR 362**	Mixed	6	66,808,707	67,379,563	5.20E-04; **1.59E-04**	
	ADG; AGE	**CNVR 602**	Mixed	11	39,893,652	40,308,946	5.02E-03; 5.20E-03	
	ADG; AGE	**CNVR 607**	Gain	11	43,760,741	43,890,386	3.44E-03; 1.48E-03	
	ADG; AGE	**CNVR 637**	Mixed	12	23,605,417	24,199,377	1.42E-03; 9.13E-04	
	ADG; AGE	**CNVR 732**	Mixed	13	206,578,011	208,240,759	**8.78E-06**; **2.43E-04**	*PFKL*
	ADG; AGE	**CNVR 852**	Gain	15	122,779,236	122,863,462	3.73E-03; 3.98E-03	
	AGE	CNVR 27	Mixed	1	68,740,759	69,522,447	5.25E-03	
	AGE	CNVR 516	Mixed	9	3,080,614	3,416,559	4.85E-03	
	AGE	CNVR 658	Gain	12	53,806,610	54,033,307	3.93E-03	*PIK3R6*, *PIK3R5*
Canadian Duroc	ADG; AGE	**CNVR 90**	Mixed	2	8,919,611	9,435,996	3.89E-03; 5.18E-03	*BSCL2*
	ADG; AGE	**CNVR 149**	Mixed	3	162,027	2,071,648	**1.51E-05**; **3.10E-05**	*GPER1*, *PDGFA*
	ADG; AGE	**CNVR 188**	Gain	3	105,163,639	105,756,806	1.87E-03; 2.73E-03	
	ADG; AGE	**CNVR 267**	Gain	4	108,348,774	108,589,640	**4.96E-04**; **4.82E-05**	
	ADG; AGE	**CNVR 277**	Gain	4	116,110,055	116,722,933	1.01E-03; 8.12E-04	
	ADG; AGE	**CNVR 354**	Loss	6	46,826,412	47,252,470	**4.05E-07**; **8.81E-07**	
	ADG; AGE	**CNVR 537**	Mixed	9	61,615,730	62,034,708	**7.70E-05**; **1.43E-04**	
	ADG; AGE	**CNVR 684**	Mixed	13	32,709,504	32,901,267	1.44E-03; 1.32E-03	*GNAI2*
	ADG; AGE	**CNVR 789**	Gain	14	121,605,565	121,639,735	**3.28E-05**; **1.90E-04**	

^1^*ADG* Average daily gain at 100 kg; *AGE* Days to 100 kg. ^2^CNVRs ID in boldface represents the CNVR identified in both traits. ^3^Gain: duplications; Loss: deletions; Mixed: Gain and Loss occurring in the same region. ^4^*P*-value in boldface: genome-wide significant; *P*-value not in boldface: suggestive significant

**Table 5:** Significant CNVRs associated with BFT in U.S. and Canadian Duroc pigs

Population	CNVR ID^**1**^	Type^**2**^	Chromosome	Start (bp)	End (bp)	***P***-value^**3**^	Candidate genes
U.S. Duroc	CNVR 69	Mixed	1	265,085,761	265,301,383	**1.09E-07**	
	CNVR 136	Loss	2	135,551,879	135,647,128	1.73E-03	
	CNVR 149	Mixed	3	162,027	2,071,648	**1.57E-05**	*GPER1*, *PDGFA*, *GNA12*
	**CNVR 152**	Mixed	3	2,789,839	3,480,462	2.94E-03	*SDK1*
	**CNVR 315**	Gain	5	52,276,104	52,737,350	4.03E-04	
	CNVR 333	Mixed	6	51,842	1,462,744	6.16E-03	
	CNVR 415	Mixed	7	29,599,648	30,265,671	4.66E-03	
	**CNVR 514**	Loss	9	619,847	1,268,010	1.52E-03	
	CNVR 584	Mixed	10	67,366,433	68,403,256	**3.74E-06**	
	CNVR 621	Mixed	11	77,144,460	78,780,052	**3.24E-07**	*GRTP1*
	CNVR 718	Gain	13	188,222,477	188,693,046	**7.95E-06**	
	**CNVR 732**	Mixed	13	206,578,011	208,240,759	3.81E-03	*PFKL*
	CNVR 807	Mixed	14	139,484,309	141,719,266	**1.64E-08**	*ADAM8*
	CNVR 862	Mixed	15	137,417,592	140,139,156	1.07E-03	
Canadian Duroc	CNVR 488	Gain	8	97,990,916	99,088,450	7.24E-03	

^1^CNVRs ID in boldface represents the CNVR had pleiotropic effects on growth and fatness traits. ^2^Gain: duplications; Loss: deletions; Mixed: Gain and Loss occurring in the same region. ^3^*P*-value in boldface: genome-wide significant; *P*-value not in boldface: suggestive significant

Analysis of growth traits identified nine suggestive (7.19E-03) and four genome-wide (3.60E-04) CNVRs associated with ADG in U.S. Duroc pigs. The candidate regions were located on SSC1, 2, 3, 5, 6, 9, 11, 12, 13, and 15. Furthermore, we also identified nine suggestive and four genome-wide CNVRs that exceeded the thresholds for association with AGE. Owing to the high genetic correlation between ADG and AGE [19], we observed 10 shared CNVRs (CNVR 83, 85, 152, 315, 362, 602, 607, 637, 732, 852) associated with both traits. In the Canadian Duroc pigs, we identified four suggestive (1.09E-02) and five genome-wide (5.43E-04) CNVRs that were significantly associated with both ADG and AGE at different *P* values. However, no CNVR was shared by the two pig populations.

Analysis of fatness traits identified eight suggestive (7.19E-03) and six genome-wide (3.60E-04) CNVRs associated with the BFT trait in U.S. Duroc pigs. Intriguingly, four CNVRs (CNVR 152, 315, 514, 732) located on SSC3, 5, 9, and 13 had pleiotropic effects on growth traits. However, we found only one suggestive (1.09E-02) CNVR that was associated with the BFT trait in Canadian Duroc pigs.

GWAS in two populations identified five CNVRs as the most significantly associated with growth and fatness traits. Additional file 5: Table S5 were summarized to reflect the phenotypic effect of the CNVRs more intuitively. In brief, pigs with increased copy numbers of CNVR 488 and 807 may have thinner backfat, and the gain type of CNVR 732, the loss type of CNVR 354 and the normal copy number of CNVR 315 may have better performance in growth traits.

Based on the data from all pigs, we further investigated the function of genes encompassing these significant CNVRs. Several common significant CNVRs that are associated with both ADG and AGE traits were found to overlap with numerous genes, and nine of these were identified as major functional candidates, including *PNPLA2*, *SDK1*, *PFKL*, and *BSCL2*. For BFT, we identified seven candidate genes, including *GPER1*, *PDGFA*, and *GRTP1*.

### Functional analysis of genes associated with trait-related CNVRs

A total of 606 genes overlapping with 31 significant CNVRs were detected based on the Ensembl [36] annotation of the *Sus scrofa* 11.1 genome (see Additional file 6: Table S6). These include 447 protein-coding genes and 110 lncRNA genes, as well as some miRNAs, small nucleolar genes (snoRNA), and processed pseudogenes. To further investigate the functional genes affecting growth performance and fatness, the Kyoto Encyclopedia of Genes and Genomes (KEGG) pathway and gene ontology (GO) analyses of protein-coding genes were carried out using the KOBAS software (version 3.0) [37].

Gene set enrichment analysis revealed many terms relevant to growth and fatness traits (see Additional file 7: Table S7, the accession numbers were obtained from Ensembl database [36]). In brief, KEGG analysis revealed that these genes mainly participate in glycosaminoglycan degradation, oxytocin signaling, and the cholinergic synapse pathway. Furthermore, GO analysis was primarily enriched in positive regulation of protein kinase B signaling, MAP kinase activity, carbohydrate metabolic process, and other important biological processes. Using information from the GeneCards database and relevant literature, we further identified several genes involved in critical pathways and biological processes (Tables 4 and 5). Here, we highlight four genes of interest that overlapped with significant CNVRs and were enriched in gene set enrichment analysis (*P* < 0.05): platelet-derived growth factor subunit A (*PDGFA*), G protein-coupled estrogen receptor 1 (*GPER1*), patatin-like phospholipase domain containing 2 (*PNPLA2*) and Bernardinelli-Seip Congenital Lipodystrophy Type 2 Protein (*BSCL2*).

## Discussion

Over the past decade, GWAS have made remarkable contributions to the discovery of common SNPs that influence complex traits [38]. However, most variants explain only a small proportion of heritability, a phenomenon known as “missing heritability” [39]. To this end, CNVs, as an important source of genetic diversity, may provide a new way for explaining the genetic variability that GWAS cannot detect [40].

In this study, we successfully identified 19,987 and 13,360 CNVs in U.S. and Canadian Duroc pigs, respectively, using rigorous criteria to reduce false-positive rates. All CNVs were merged to generate 953 CNVRs in the two populations, accounting for ~ 10.90% of the pig autosomal genome (*Sus scrofa* 11.1). The results showed that the size and frequency of duplications were much higher than those of deletions in the large fragment (> 10 kb) CNVs (27,944 gains vs. 5403 losses). Previous CNV studies reported similar cases. For example, a CNV study conducted by Long et al. [41] using Porcine SNP60 BeadChip, identified approximately 70.6% duplications and 29.4% deletions. Using Next-generation sequencing data, Zheng et al. [17] also reported that the frequency of duplications was higher than that of deletions in the Duroc and Meishan pigs. This phenomenon suggests that although CNVs can cause duplications or deletions at the same locus in different populations [42], the genome is more tolerant to duplications than it is to deletions [43], and these duplications are more likely to occur in large CNVs (> 10 kb) [5, 44]. In addition, based upon SNP chip design principles, it can be inferred that if there are more than 2 copies (duplications) in a diploid organism, then the likelihood of identifying a high frequency SNP and the chance of detecting variation may be greater than if there are only 0, 1 or 2 copies [45].

To evaluate the accuracy of the PennCNV software in identifying CNVs, we performed qPCR validation for nine randomly selected CNVRs and successfully confirmed seven of these (~ 77.8%). This percentage is similar to that reported by Wang et al. [28] (75%), Dong et al. [46] (70%), and Wang et al. [33] (80%). We also observed that two “failed” CNVRs were inconsistent with our expectations. Multiple factors may have contributed to the discordance in the results. For example, the sparse probes on the SNP chip may cause the estimated size of CNVRs to be larger than their actual length. Consequently, the primers may have been designed outside the exact boundaries of the CNVRs [46]. Additionally, these results indicate that a high proportion of singleton CNVs exists in the population [47].

We also compared our results with those of previous studies on CNVRs and found a low overlap rate [17, 27–34]. In brief, a total of 465 CNVRs entirely or partially overlapped with previously reported CNVRs. A considerable overlap rate was observed with the results reported by Zheng et al. [17], whereas those reported by Xie et al. [31] gave the lowest overlap. These discrepant observations may be due to differences in the breeds studied. In this study, the large number of samples used for CNV detection led us to identify more novel CNVRs than previous studies. It also suggests that a vast number of CNVs in the pig genome have not been discovered [48]. In addition, most of the previous studies were based on the Sscrofa10.2 genome version, whereas the comparative work in our study was based on version Sscrofa11.1. Thus, based on the vast differences between these two genome versions [49], many CNVRs in Sscrofa10.2 could not be successfully converted to Scrofa11.1 (Table 2). Differences in SNP density after quality control, as well as different CNV detection platforms, algorithms, and criteria for CNV determination could also explain this outcome [33]. Intriguingly, even within the same breed, different genetic backgrounds may have significant effects on reproducibility. In our previous GWAS, principal component analysis (PCA) and linkage disequilibrium (LD) decay analysis suggested that the U.S. Duroc population had a genetic background that differed from that of the Canadian Duroc population [50, 51]. As shown by our results, only 348 of 953 CNVRs were detected in both populations. In addition, population size might also affect CNV detection. In our study, the number of U.S. Duroc pigs used for CNV detection was 1.3 times higher than that of Canadian Duroc pigs (3770 vs. 2857), which may have led to differences in the final numbers of CNVs (19,987 vs. 13,360) and CNVRs (802 vs. 499).

Although CNVs are widespread in pigs and are associated with economically important traits, the full relevance of CNVs to the genetic architecture of growth rate and fatness across all stages is yet to be elucidated. To further investigate the relationship between CNVs and complex traits (ADG, AGE, and BFT), we performed CNVR-based GWAS on these two pig populations. We identified 16 significant CNVRs that were associated with ADG or AGE in U.S. Duroc pigs, including 10 CNVRs that were significant for both traits. A similar pattern was observed in the Canadian Duroc pigs. For instance, we detected nine CNVRs that affect both traits. The computational formula of the adjusted ADG was inversely proportional to that of the adjusted AGE in this study, and both traits also had a relatively high genetic association [19]. This may explain why most CNVRs were significant for both traits.

However, the results of GWAS between U.S. and Canadian Duroc pigs differed substantially, and we found no shared CNVRs when we analyzed ADG and AGE in the two populations. Moreover, we detected 14 BFT-related CNVRs in U.S. pigs, but only one was identified in the Canadian population. This finding highlights the complex genetic architecture of growth and fatness traits. Although Duroc is considered a single breed, substantial genetic differences exist between subpopulations [52], as shown for the U.S. and Canadian Duroc pigs in this study. These results are consistent with those of Zhou et al. [50] and Zhuang et al. [51]. It is presumed that, due to differences in natural and human selective pressures, genetic drift and the exchange of genetic material has resulted in less consistency in CNVRs between the two populations [53]. Therefore, genetic differentiation between the two populations may have a substantial impact on the genome localization of genetic variants [51]. More notably, four CNVRs—CNVR 152 (SSC3: 2.8–3.5 Mb), CNVR 315 (SSC5: 52.3–52.7 Mb), CNVR 514 (SSC9: 0.6–1.3 Mb), and CNVR 732 (SSC13: 206.6–208.2 Mb)—were associated with growth and fatness traits in U.S. Duroc population. These results suggest that these CNVRs may play a pleiotropic role in regulating pig growth and fat deposition [18, 20].

To better understand the molecular function of the genes involved in significant CNVR, we examined their GO and KEGG classification. Many of these genes participated in carbohydrate metabolic process, MAP kinase activity, glycosaminoglycan degradation, and O-glycan biosynthesis. Consequently, we highlighted four genes; *PDGFA*, *GPER1*, *PNPLA2*, and *BSCL2*, which were previously recognized as important for body weight and fat deposition, but their roles in pigs are poorly understood. White adipose tissue is recognized as an energy-storing organ that is closely associated with fat deposition and body weight [54]. Gonzalez et al. [55] found that *PDGFA* plays a vital role in the proliferation and maintenance of adipocyte progenitors in dermal adipose tissue through the PI3K-Akt pathway. Previous studies also reported that *PDGFRα* is activated by the homodimers *PDGFA*, *PDGFB*, and *PDGFC*, whereas *PDGFRβ* is activated by *PDGFB* and *PDGFD* [56, 57]. More importantly, human adipose tissue differentiation into beige or white adipocytes depends on *PDGFRα/PDGFRβ* signaling [58]. The *BSCL2* gene also participates in adipocyte differentiation and lipid droplet formation. Mutations in the *BSCL2* gene cause human congenital lipodystrophy, an autosomal recessive genetic disease characterized by almost complete loss of adipose tissue, insulin resistance, and fatty liver [59, 60]. The gene *GPER1* encodes G protein-coupled estrogen receptor 1, which is involved in metabolism and immunity [61]. Sharma et al. [62] reported that weight gain in male *GPER*-knockout (KO) mice was associated with visceral and subcutaneous fat. However, these *GPER* KO mice showed no differences in food intake or exercise activity levels compared with wild-type littermates. This observation demonstrates that *GPER* may regulate metabolic parameters associated with obesity. As an important triglyceride hydrolase in mammalian cells, *PNPLA2* predominantly performs the first step in triglyceride hydrolysis. Dai et al. [63] revealed that functional polymorphisms in the 5′ upstream region of *PNPLA2* are potential DNA markers for backfat thickness in Duroc pig breeding programs.

In recent years, studies on the influence of CNVs on complex traits have gradually been thrust into the limelight [17, 33]. To the best of our knowledge, the present study represents the largest sample size used for the detection of genome-wide CNVs in Duroc pigs. However, due to the sparse markers in the SNP chip used, we may have overestimated the frequency of large-scale CNVs detected in our study. Accordingly, high-density SNP chips or whole-genome sequencing technologies should be applied for further CNV detection.

## Conclusions

In this study, we performed genome-wide CNV detection and CNVR-based GWAS for growth and fatness traits in a large population of U.S. and Canadian Duroc pigs. A total of 953 CNVRs were detected in these two populations, accounting for ~ 10.90% of the pig autosomal genome. Moreover, 35 CNVRs were associated with growth and fatness traits. However, we found no shared CNVR QTL in the two populations among these CNVRs. These findings indicate that genetic differences between the two populations may have a substantial impact on the genomic localization of genetic variants. We also identified major candidate genes, including *PDGFA*, *GPER1*, *PNPLA2*, and *BSCL2*, that may be related to growth and fatness traits. Our results provide valuable insights into the genetic mechanisms underlying growth and fatness traits in pigs.

## Methods

### Ethics statement

The animals and experimental methods used in this study follow the guidelines of the Ministry of Agriculture of China and the Use Committee of South China Agricultural University (SCAU). The ethics committee of SCAU (Guangzhou, China) approved all animal experiments. The experimental animals were not anesthetized or euthanized in this study.

### Samples and phenotype data

Experimental animals were raised at the Wens Foodstuff Group Co., Ltd. (Guangdong, China) of Duroc core breeding farms. A total of 6627 Duroc pigs were used, including 3770 (2280 males and 1490 females) Duroc pigs of U.S. origin and 2857 (1570 males and 1287 females) Duroc pigs of Canadian origin, born between 2013 and 2018. Once these 6627 Duroc pigs reached a body weight of 30 ± 5 kg, they were transferred to the test station. During the experiment, all pigs were raised under normal management conditions, provided with drinking water, and were freely fed. Additionally, data on average daily gain at 100 kg (ADG), days to 100 kg (AGE), and backfat thickness at 100 kg (BFT) were collected from each population; a more detailed description of the phenotypic measures can be found in our previous publication [50]. In brief, when their body weight reached approximately 100 kg (100 ± 5 kg), ADG and AGE traits were measured and adjusted to 100 kg. The adjusted formula for AGE is as follows [19, 50]:

$$ AGE\  adjusted\ to\ 100\  kg\ (day)= Measured\  age-\left(\frac{Measured\ weight-100\  kg}{Correction\ factor\  one}\right) $$

where correction factor one differs between sire and dam based on the formulas below:

$$ Sire: Correction\ factor\  one=\frac{ Measured\ weight}{Measured\  age}\times 1.826 $$

$$ Dam: Correction\ factor\  one=\frac{ Measured\ weight}{Measured\  age}\times 1.715 $$

The following formula was used for adjusted ADG [19, 50]:

$$ ADG\  adjusted\ to\ 100\  kg\ \left( kg/ day\right)=\frac{100\  kg}{AGE\  adjusted\ to\ 100\  kg} $$

In addition, when their body weight reached 100 ± 5 kg, the BFT phenotype was measured using an Aloka 500 V SSD B ultrasound probe (Corometrics Medical Systems, USA) from the 10th-rib to the 11th-rib of the pig [64]. Adjusted 100 kg BFT was obtained from the Canadian Centre for Swine Improvement (http://www.ccsi.ca/Reports/Reports_2007/Update_of_weight_adjustment_factors_for_fat_and_lean_depth.pdf) using the following formula:

$$ BF\  adjusted\ to\ 100\  kg\ (mm)= Measured\ backfat\ thickness\times Correction\ factor\  two $$

where $Correction\ factor\  two=\frac{A}{A+\left[B\times \left( Measured\ Weight-100\right)\right]}$, A = 13.468 and B = 0.111528 in sires, and A = 15.654 and B = 0.156646 in dams. Before the association analysis, outliers outside the mean ± 3 standard deviations were removed.

### SNP genotyping and quality control

Genomic DNA was extracted from ear tissue using the traditional phenol/chloroform method, and the quality of DNA in all samples (6627 DNA samples) was assessed based on light absorption ratio (A_260/280_ and A_260/230_) and gel electrophoresis, using a DNA concentration of 50 ng/μL [65]. Samples were genotyped using the Illumina GeneSeek 50 K SNP array (Neogen, Lincoln, NE, United States) with 50,649 SNP markers across the entire genome. Quality control was performed using the PLINK software v1.90 [66]. SNPs located in sex chromosomes, or without positional information, were discarded and only samples with high-quality genotyping (call rate of 90% and above) were retained [27, 41, 67]. Finally, a set of 46,458 informative SNPs from 3770 Duroc pigs of U.S. origin and 46,458 informative SNPs from 2857 Duroc pigs of Canadian origin was used for CNV detection.

### CNV detection

The PennCNV software v1.0.5 was used to identify individual-based CNVs by combining the SNP signal data of log R ratio (LRR) and B allele frequency (BAF) as well as the population frequency of the B allele (PFB). The LRR and BAF values for each SNP were computed using the GenomeStudio software (v2.0; Illumina, Inc., USA). The Perl comppile_pfb.pl command in PennCNV was used to calculate PFB based on the BAF of each SNP. Moreover, the wave adjustment procedure was conducted using the -gcmodel option in the PennCNV to reduce the impact of genomic waves [68]. We calculated the GC content in the 500 kb genomic region around each SNP derived from the Sscrofa 11.1 version of the pig reference genome (http://ensemble.org/Sus_scrofa/Info/Index). PennCNV was run using the -*test* option without considering pedigree information, as the relationship among the pigs in our study population is unknown. The final CNVs were identified by retaining high-quality samples according to the following criteria: LRR < 0.3, BAF drift < 0.01, and GC wave factor of LRR < 0.05. Meanwhile, to further decrease false-positive CNV calls, CNVs with consecutive SNPs ≥3 and CNV length ≥ 10 kb were retained. We also used the BEDTools software v2.26.0 [69] to merge CNVs with at least 1 bp overlap in all samples to define the CNV region (CNVR) [17]; CNV and CNVR borders were determined based on the location of SNP markers. The CNVRuler software v1.3.3.2 [70] was used to define three types of CNVR: loss, gain and mixed (gains and losses occurring in the same region). In addition, we matched CNVs with the corresponding CNVR in each population to obtain the CNVRs. In other words, CNVRs with full coverage CNV sequences were considered population-based CNVRs. A final set of 802 CNVRs mapped in 3303 U.S. Duroc pigs and 499 CNVRs mapped in 2677 Canadian Duroc pigs was used for subsequent analyses.

### Quantitative PCR validation

We chose real-time quantitative polymerase chain reaction (qPCR) to validate the CNVRs detected by PennCNV. A total of nine CNVRs were randomly selected based on the CNVR type (loss, gain, and mixed) and frequency in the population. Due to uncertainty in the boundaries of the identified CNVRs, we used the Oligo 7 software [71] to design primers for specific regions in the *ELFN1*, *PUSL1*, *MAPRE2*, *SGMS2*, *PCID2*, *DSCAM*, *GATD3A ADGRA1*, and *LIFR* genes (see Additional file 4: Table S4). We also selected the *GCG* gene as the reference locus because this gene is highly conserved among pigs and exists as a single copy in the reference genome [17, 33, 72]. A total of 74 DNA samples were randomly selected for qPCR validation, and normal samples identified with no copy number change in the test region were used as references. Real-time quantitative PCR was conducted using Qiagen’s Quantitative Reaction Kit (QuantiFast SYBR Green PCR kit, Qiagen, Hilden, Germany). The PCR reaction was performed using a total 10 μL volume consisting of the following reagents: 1 μL DNA (50 ng/μL), 0.3 μL of both forward and reverse primers (10 pM/μL), 5 μL Blue-SYBR-Green mix (2×), and 3.4 μL water. The PCR conditions were as follows: 95 °C denaturation, hot start 5 min; 45–50 PCR cycles (95 °C, 10 s, 60 °C, 15 s, and 72 °C, 20 s); dissolution curve (95 °C, 15 s, 55 °C, 15 s, and 95 °C, 15 s). All reactions were carried out on 384-well clear reaction plates, and each sample was amplified in triplicate, with average *C*_*t*_ values calculated for further copy number determination. The relative copy number difference in the test region was determined using 2 × 2^-ΔΔ*Ct*^, where ΔΔ*C*_*t*_ = [(mean *C*_*t*_ of the target gene in the test sample) - (mean *C*_*t*_ of *GCG* in the test sample)] - [(mean *C*_*t*_ of the target gene in the reference sample) - (mean *C*_*t*_ of *GCG* in the reference sample)] [73]. Values of approximately 2 were considered normal. A value of 3 or more and a value of 1 or less represented gain and loss statuses, respectively.

### CNVR genotyping and GWAS

To provide the required input for GWAS, specific genotyping for CNVR was necessary. We used in-house script to genotype CNVRs in U.S. and Canadian Duroc pigs into “+/+”, “+/−”, “−/−”, following previous studies [74, 75].

In this study, the GEMMA software v0.98.1 [76] was applied to a univariate linear mixed model to conduct GWAS on a single population. To improve the accuracy of the GWAS results, we filtered the CNVR datasets with frequencies lower than 0.5% in each population [32]. A final set of 139 CNVRs in 3303 U.S. Duroc pigs and 92 CNVRs in 2677 Canadian Duroc pigs was selected for association analysis. Before GWAS, genomic relatedness matrix (GRM) and principal component analysis (PCA) based on SNP datasets for each population were generated using the GEMMA and GCTA software v1.92.4beta [77]. The statistical model used was as follows:

$$ y= W\alpha + X\beta +u+\varepsilon $$

where *y* represents a vector of the corrected phenotypic value for each population; *W* is the incidence matrix of covariates, including fixed effects of the top three eigenvectors of PCA, sex, birth weight, and parity; *α* represents the vector of corresponding coefficients including the intercept; *X* is the vector of CNVR marker genotypes; *β* specifies the corresponding effect size of the CNVR; *u* is the vector of random effects, with *u*~*MVN*_*n*_ (0, *λτ*^−1^*K*); *ε* is the vector of random residuals, with *ε*~*MVN*_*n*_ (0, *τ*^−1^*In*); *λ* signifies the ratio between two variance components; *τ*^−1^ is the variance of the residual errors; *K* is GRM; *I* is an n × n identity matrix; *MVN*_*n*_ denotes the n-dimensional multivariate normal distribution. In the CNVR-based GWAS, the Bonferroni method was used to determine the genome-wide significant (0.05/N) threshold, where N represents the number of CNVRs. Given that is a stringent criterion, a more lenient threshold was also used for detecting the suggestive (1/N) CNVRs [78, 79].

### Candidate gene identification and functional enrichment analysis

The physical position information was obtained from the Sscrofa 11.1 version of the pig reference genome. Genes that overlapped with significant CNVRs were selected for KEGG pathway and GO analyses using KOBAS v3.0. Enriched terms with *P* < 0.05 based on Fisher’s exact test were selected for further exploration of the genes involved in biological pathways and processes [65, 80]. GeneCards (http://www.genecards.org/) and Ensembl (www.ensembl.org/biomart/martview) were used to query gene functions.

## Supplementary Information

**Additional file 1: Table S1.** Overview of CNVRs for two Duroc populations.

**Additional file 2: Table S2.** Information of CNVRs shared by two Duroc populations.

**Additional file 3: Table S3.** Identified CNVRs compared with previous studies.

**Additional file 4: Table S4.** qPCR primer and probe sequence information.

**Additional file 5: Table S5.** The phenotypic effect of the most significant CNVRs in U.S. and Canadian Duroc pigs.

**Additional file 6: Table S6.** Information of genes in the significant CNVRs.

**Additional file 7: Table S7.** KEGG and GO Enrichment Analysis for significant CNVRs gene set by KOBAS.

## Data Availability

The SNP genotyping data containing variant information for the U.S. (*n* = 3,770) and Canadian (*n* = 2,857) Duroc pigs are not publicly available because the genotyped animals belong to commercial breeding companies, but they can be obtained from the corresponding author under reasonable requirements. Pig genome (*Sus scrofa* 11.1), annotations (v103) and the accession numbers listed in Table S6 and Table S7 can be obtained from ENSEMBL (http://ftp.ensembl.org/pub/release-103/).

## Abbreviations

CNV: Copy number variation
CNVR: Copy number variation region
CN: Copy number
ADG: Average daily gain
AGE: Days to 100 kg
BFT: Backfat thickness
SSC: *Sus scrofa* chromosome
GWAS: Genome-wide association study
SNP: Single nucleotide polymorphism
INDEL: Insertion and deletion
QTL: Quantitative trait locus
qPCR: Quantitative polymerase chain reaction
