# Supplementary material for: Genome-wide detection of CNV regions and their potential association with growth and fatness traits in Duroc pigs
Source: BMC Genomics. 2021 May 8;22:332. doi: 10.1186/s12864-021-07654-7 (PMC8106131; doi:10.1186/s12864-021-07654-7)
Supplement: Supplementary file 5 — Additional file 5: Table S5. The phenotypic effect of the most significant CNVRs in U.S. and Canadian Duroc pigs. [file 12864_2021_7654_MOESM5_ESM.docx]

**Additional file 5: Table S5** **The phenotypic effect of the most significant CNVRs in U.S. and Canadian Duroc pigs**

| **Population** | **Traits^1^** | **CNVR ID** | **Type^2^** | ***P*-value** |  | **Genotype^3^** |  |
| --- | --- | --- | --- | --- | --- | --- | --- |
|  |  |  |  |  | **Gain** | **Loss** | **Normal** |
| U.S. Duroc | ADG | CNVR 732 | Mixed | 8.78E-06 | 624.99 ± 22.93(26) | 601.41 ± 33.97(59) | 619.65 ± 31.69(3,218) |
|  | AGE | CNVR 315 | Gain | 4.78E-05 | 160.09 ± 8.49(177) | / | 158.93 ± 8.19(3,126) |
|  | BFT | CNVR 807 | Mixed | 1.64E-08 | 8.74 ± 0.91(918) | 9.28 ± 1.15(127) | 8.94 ± 0.94(2,258) |
| Canadian Duroc | ADG | CNVR 354 | Loss | 4.05E-07 | / | 639 ± 45.3(92) | 610.94 ± 41.72(2,585) |
|  | AGE | CNVR 354 | Loss | 8.81E-07 | / | 154.07 ± 11.27(92) | 161.39 ± 11.06(2,585) |
|  | BFT | CNVR 488 | Gain | 7.24E-03 | 8.14 ± 1.3(18) | / | 9.56 ± 1.77(2,659) |

^1^ADG: Average daily gain at 100 kg; AGE: days to 100 kg; BFT: Backfat thickness at 100 kg; ^2^Gain: duplications; Loss: deletions; Mixed: Gain and Loss occurring in the same region; ^3^Genotype include Gain, Loss and Normal (normal copy in this region) type of CNVRs in population, the data in the table represent the mean ± standard deviation (Number of animals).
